# The Implications of Metabotypes for Rationalizing Therapeutics in Infants and Children

**DOI:** 10.3389/fped.2015.00068

**Published:** 2015-07-29

**Authors:** Theodora Katsila, George P. Patrinos

**Affiliations:** ^1^Department of Pharmacy, School of Health Sciences, University of Patras, Patras, Greece

**Keywords:** pharmacometabolomics, metabotypes, pharmacogenomics, neonatology, pediatrics

## Introduction

Nowadays, new “omics” disciplines aim to shed light on post-genomic activity and/or monitor biological functions of interest at various levels of cellular organization. Each one of these holistic approaches exhibits its own power as well as limitations.

Pharmacogenomics focuses on individual drug variability aiming to optimize drug efficacy and minimize toxicity (1). In the context of pharmacogenomics, though, it has been stated that neither physiological nor environmental influences on drug pharmacokinetics are considered. Metabolomics has been introduced to meet this exact need as metabolites provide a global interpretation of biological effects (2). Metabolite identification in an individual’s body fluids is often associated to an overall understanding of metabolic dynamics per condition of interest. As such, metabolomic studies have a great potential toward the prediction or evaluation of drug metabolism, permitting continued treatment with the optimal drug or dosage based on the variations in drug metabolism and ability to respond to treatment (3, 4). The alteration in an individual’s metabolomic profile associated with therapy (pharmacometabolomics) is expected to be one of the hallmarks of precision medicine, as the metabotype – the metabolic profile of an individual – can assist toward a more accurate definition of drug response and even disease heterogeneity.

In pediatrics and neonatology, drug pharmacokinetic properties differ substantially from those in adults (in particular, during the first 4 years of a newborn’s life) (5). Hence, it is rather difficult to obtain an effective and at the same time safe pediatric dose via a linear reduction of the adult dose. Even today, this consists a great challenge as several drugs are not specifically approved for pediatric use, and thus, a dosage approximation becomes necessary. Undoubtedly, there is a great potential for metabolomic studies to rationalize therapeutic use in children (Table 1).

**Table 1:** **A synopsis of (pharmaco)metabolomics studies**.

Topics	Year	Reference
The composition of human milk	1979	(13)
Children with autism are differentiated from their unaffected siblings and age-matched controls by urinary metabolic phenotyping	2010	(17)
Metabolomic analysis of bronchoalveolar lavage fluid in preterm infants that suffer from respiratory distress syndrome	2011	(11)
Metabolomics and Patent Ductus Arteriosus diagnosis	2011	(7)
The metabolomic profiling of children’s brains upon general anesthesia with sevoflurane and propofol.	2012	(18)
Celiac disease autoimmunity in genetically at risk infants, gluten exposure and proof of concept of microbiome–metabolome analysis	2012	(19)
The study of urinary organic acids in respiratory chain deficiencies in children	2012	(20)

## Applications in Neonatology

It is true that in-depth clinical research is lacking in such a vulnerable population as neonates. Thus, the implementation of (pharmaco)metabolomics in neonatology is expected to play a key role in order to: (i) predict the outcome of drug treatment, (ii) monitor the drug-related alterations in metabolic pathways, and (iii) identify the metabolites (and their levels) following drug administration. Recently, both metabolomic and pharmacometabolomic studies have been focused on a range of aspects: inborn errors of metabolism, respiratory distress syndrome, patent ductus arteriosus, drug treatment and even maternal milk.

Patent ductus arteriosus is one of the most common congenital heart defects that preterm infants suffer from, being associated with mutations in the germ cell line as a result of cumulated cell replications and consequently, advanced paternal age (6). Atzori et al. have employed a ^1^H NMR-based metabolomic analysis of the first urine passed by term and preterm infants at early life (3–4 days) and discriminated among the term- and preterm-infants without PDA and preterm infants with persistent patent ductus arteriosus (7). Notably, the authors were also able to define responder and non-responder phenotypes to ibuprofen. This study implies that pharmacometabolomics may serve as an early diagnostic and monitoring means for this defect, avoiding unnecessary drug prophylaxis.

Respiratory distress syndrome is considered one of the important causes of morbidity and mortality in preterm newborns, as it is often characterized by severe complications due to endotracheal intubation and mechanical ventilation, which leads to lung tissue damage and surfactant inactivation (8–10). The effects of surfactant therapy in preterm infants as well as their lung status were investigated by Fabiano et al. (11). For this, serial bronchoalveolar lavage fluid samples were collected at birth prior to surfactant instillation, post-surfactant instillation during mechanical ventilation, and at extubation time-points and were analyzed by ^1^H NMR spectroscopy and GC-MS (11). The analyses resulted in the identification of 25 metabolites (10 of known molecular structure) that were overexpressed during mechanical ventilation, following surfactant instillation. Metabolite identification, in this case, could promote biomarker discovery for the respiratory distress syndrome in preterm infants.

According to the World Health Organization guidelines, neonates should be exclusively breast fed for the first 6 months of life (12). Human breast milk contains nutrients as well as several bioactive components (growth factors, antimicrobial compounds, immune-enhancing molecules) (13). However, there are claims that breast milk alone is incapable of meeting the highly demanding nutritional needs of preterm and low body weight infants (14, 15). As an alternative, formula milk has been introduced, although its use is still in debate. It is true that formula milk is enriched with all the nutrients that neonates need plus iron and fatty acids to promote brain development. Nonetheless, formula milk is produced according to standard recipes without considering the inherent individuality of development that makes each preterm infant different from others. A ^1^H NMR-based metabolomic study was performed to determine the composition of breast milk from mothers of preterm and low body weight neonates during the first month of lactation. Significant qualitative and quantitative differences were obtained, when human breast milk was compared to formula milk. Notably, breast milk exhibited a great degree of biochemical variability (16).

## Applications in Childhood

Autism appears to be an excellent example. This is an early onset developmental disorder, characterized by a severe life-long impact on both behavior and social functioning, with associated metabolic and gastrointestinal abnormalities whose etiology is still unclear (5). Yap et al. investigated the urinary metabolic phenotypes of children aged 3–9 years (39 autistic subjects, 28 non-autistic siblings, and 34 age-matched healthy subjects) using ^1^H NMR spectroscopy and pattern recognition methods (17). Findings in autistic children suggested a perturbation in the tryptophan-nicotinic acid metabolic pathway, sulfur, and amino acid metabolism. The additional biochemical alterations found in autistic children were consistent with some abnormalities of gut microbiota (17). The latter could be used toward the monitoring of therapeutic interventions.

The induction of anesthesia by either inhalation of sevoflurane or intravenous propofol is common. For this, a ^1^H NMR spectroscopy-based metabolomic profiling was applied to characterize the cerebral metabolic status of 59 children, undergoing NMR imaging, after approximately 60 min of anesthesia (18). Upon sevoflurane administration, higher glucose and lactate levels were obtained, indicating a greater neuronal activity.

Celiac disease is a rather unique autoimmune disorder as both genetic factors (HLA class II genes DQ2 and/or DQ8) and environmental influences (gluten) are known to contribute, but yet not sufficient for disease development. A thorough study by Sellitto et al. defined (i) how the microbial communities that colonize infants change from birth to 24 months, (ii) the impact of early vs. late gluten introduction on the gut microbiota and metabolome, and (iii) the switch from gluten tolerance to immune response (19). Notably, the microbiota of newborns being genetically predisposed for the disease exhibited great differences, when compared to those from newborns with a non-selected genetic background. According to the retrospective analyses of gastrointestinal microbiota and metabolomic findings, candidate predictive biomarkers for autoimmune development in subjects genetically at risk might be identified. If so, pharmaceutical interventions would be possible during the pre-clinical phase of the disease to prevent the onset of the disease.

A recent study investigated 39 children with defined respiratory chain deficiencies, revealing the presence of 255 endogenous and 46 exogenous substances (20). Among them, 24 metabolites were identified, being highly and statistically significant for the combined and clinically related group of respiratory chain deficiencies. Consequently, a metabolite profile was obtained having the potential to define a characteristic signature for respiratory chain deficiencies. This may enable the development of a non-invasive screening method for respiratory chain deficiencies (20).

## Conclusion and Future Perspectives

A great potential is anticipated for metabolomics to guide therapeutic choices in children, as dosage approximation is often inevitable. We and others feel that the metabolic profile of a pediatric patient at baseline and prior to treatment will reveal information on individual drug response and possibly, disease heterogeneity. This is why the holistic approach of pharmacometabolomics is expected to have a fundamental impact in the future, enabling our in-depth understanding of individual variations in drug response phenotypes as well as the design of individualized therapeutic approaches via metabotypes’ prediction. The interplay of pharmacogenomics, metabolomics, and pharmacometabolomics is rather intriguing, as the identification of the genetic components of the metabolome seems to be the key, particularly during the first 4 weeks of a newborn and during childhood. At the same time, “pharmacometabolomics-informed pharmacogenomics” (21) is expected to give a new insight and contribute to efforts for personalized medicine into pediatrics and neonatology.

## Conflict of Interest Statement

The authors declare that the research was conducted in the absence of any commercial or financial relationships that could be construed as a potential conflict of interest.
